# Ticks and Tick-Borne Diseases in Central America and the Caribbean: A One Health Perspective

**DOI:** 10.3390/pathogens10101273

**Published:** 2021-10-02

**Authors:** Roxanne A. Charles, Sergio Bermúdez, Pavle Banović, Dasiel Obregón Alvarez, Adrian Alberto Díaz-Sánchez, Belkis Corona-González, Eric Marcel Charles Etter, Islay Rodríguez González, Abdul Ghafar, Abdul Jabbar, Sara Moutailler, Alejandro Cabezas-Cruz

**Affiliations:** 1 Department of Basic Veterinary Sciences, School of Veterinary Medicine, Faculty of Medical Sciences, University of the West Indies, St. Augustine, Trinidad and Tobago; 2 Department of Medical Entomology, Gorgas Memorial Institute for Health Research, Panama 0816-02593, Panama; sbermudez@gorgas.gob.pa; 3 Ambulance for Lyme Borreliosis and Other Tick-Borne Diseases, Pasteur Institute Novi Sad, 21000 Novi Sad, Serbia; pavle.banovic.mf@gmail.com; 4 Department of Microbiology with Parasitology and Immunology, Faculty of Medicine, University of Novi Sad, 21000 Novi Sad, Serbia; 5 School of Environmental Sciences, University of Guelph, Guelph, ON N1G 2W1, Canada; dasielogv@gmail.com; 6 Department of Biology, University of Saskatchewan, 112 Science Place, Saskatoon, SK S7N 5E2, Canada; adiasanz88@gmail.com; 7 Department of Animal Health, National Center for Animal and Plant Health, Carretera de Tapaste y Autopista Nacional, Apartado Postal 10, San José de las Lajas, Mayabeque 32700, Cuba; bcorona@censa.edu.cu; 8 CIRAD, UMR ASTRE, Petit-Bourg, 97170 Guadeloupe, France; eric.etter@cirad.fr; 9 ASTRE, University de Montpellier, CIRAD, INRAE, 34398 Montpellier, France; 10 Department of Mycology-Bacteriology, Institute of Tropical Medicine Pedro Kourí, Marianao 13, Havana 10400, Cuba; Islay@ipk.sld.cu; 11 Department of Veterinary Biosciences, Melbourne Veterinary School, the University of Melbourne, Werribee, VIC 3030, Australia; abdul.ghafar@unimelb.edu.au (A.G.); jabbara@unimelb.edu.au (A.J.); 12 Anses, INRAE, Ecole Nationale Vétérinaire d’Alfort, UMR BIPAR, Laboratoire de Santé Animale, 94700 Maisons-Alfort, France; sara.moutailler@anses.fr

**Keywords:** ticks, tick-borne diseases, Central America, Caribbean, One Health

## Abstract

Ticks have complex life cycles which involve blood-feeding stages found on wild and domestic animals, with humans as accidental hosts. At each blood-feeding stage, ticks can transmit and/or acquire pathogens from their hosts. Therefore, the circulation of tick-borne pathogens (TBPs), especially the zoonotic ones, should be studied in a multi-layered manner, including all components of the chain of infections, following the ‘One Health’ tenets. The implementation of such an approach requires coordination among major stakeholders (such as veterinarians, physicians, acarologists, and researchers) for the identification of exposure and infection risks and application of effective prevention measures. In this review, we summarize our current knowledge on the epidemiology of tick-borne diseases in Central America and the Caribbean and the challenges associated with the implementation of ‘One Health’ surveillance and control programs in the region.

## 1. Introduction

The global prevalence of ticks and tick-borne diseases (TBDs) has increased in human and animal populations, possibly due to changes in human behavior (e.g., increased outdoor activities), demographics, climate, and land utilization, resulting in the emergence and re-emergence of infectious and zoonotic diseases [1,2]. Particularly, there is a growing body of evidence suggesting that the occurrence of TBDs is on the rise as a consequence of climate change, since the latest data released by the U.S. National Climate Assessment (NCA4) suggest a 2 °C increase in annual average temperature—in line with the mid-century (2036–2065)—which will cause an increment by over 20% of the number of Lyme disease cases in the U.S. in the coming decades [3]. Ticks and wildlife are the key reservoirs of tick-borne pathogens (TBPs) which cause a wide spectrum of TBDs, including anaplasmosis, babesiosis, borreliosis, ehrlichiosis, and rickettsioses in both humans and animals. These TBDs are distributed globally and affected areas include subtropical and tropical regions such as Central America and the Caribbean (CAC) [4]. Information on various important aspects of TBDs in the CAC is limited. Therefore, expanded research on the epidemiology, ecology, and diagnosis of TBPs in animals and humans in this region is needed. Future research projects in the area of TBDs should consider active collaborations between human and veterinary doctors (i.e., a ‘One Health’ approach).

Since the recognition of similar diseases affecting humans and animals, the concept of ‘One Health’ has been applied for more than two centuries [5]. This approach was revived during the outbreak of severe acute respiratory syndrome (SARS) in early 2003, followed by the highly pathogenic avian influenza virus (H5N1) [6]. Based on the definition by the ‘One Health’ Global Network: “*One Health recognizes the interconnection among the health of humans, animals and ecosystems which involves the application of a coordinated, collaborative, multidisciplinary and cross-sectoral approach to address potential or existing risks that originate at the animal-human-ecosystem interface*” [6]. This concept can also be applied to the study of epidemiology of TBDs [7,8,9]. Wild and domestic animals infested by ticks play an integral role in the epidemiology of zoonotic TBDs. For example, the tropical bont tick, *Amblyomma variegatum*, is a known vector of *Ehrlichia ruminantium* (etiological agent of heartwater) in ruminants, and also of *Rickettsia africae* (causative agent of tick-bite fever in humans) and *Rickettsia conorii* (causative agent of boutonneuse fever) in humans [10,11,12]. Thus, if a livestock farmer and the animals on a farm are bitten by infected ticks, the risk of contracting a TBD is plausible. Assuming that the farmer seeks veterinary care for cattle affected by heartwater, the veterinarian has the responsibility to treat the animal, recommend treatment of the environment and advise the owner about the potential risks of being bitten by these ticks. Conversely, if the farmer seeks medical attention for an unexplained febrile illness after being bitten by a tick, the physician should enquire about the animals and advise the farmer to seek the aid of a veterinarian. Discussions between veterinarians and physicians should also be held to identify the vector, clinical signs, diagnosis, and treatment protocols in a timely manner. These scenarios with frequent and effective communication between veterinarians and physicians are ideal but rarely executed in real life. In regions such as the CAC, where veterinary and human healthcare systems are limited, the establishment and implementation of ‘One Health’ approaches may be challenging. In this review, we have summarized our current knowledge on the epidemiology of TBDs in the CAC as well as the current challenges to implementing ‘One Health’ surveillance and control programs in the region.

## 2. Overview of Ticks and Tick-Borne Pathogens in the CAC

### 2.1. Ticks and Tick-Borne Bacterial and Protozoal Diseases in Animals in the CAC

Central America and the Caribbean region encompass a vast biodiversity of fauna, including ticks, with approximately 80 species reported to date [13,14,15,16,17]. Despite the fact that TBDs have been reported in most countries in the CAC region, more epidemiological studies are needed to determine the ecology and prevalence of ticks and TBDs in the animal population of the region [4,18]. Ticks and TBPs affecting animals in the CAC are summarized in Table 1.

The most prevalent TBPs and diseases reported in companion animals, especially dogs, include tropical canine pancytopenia (caused by *Ehrlichia canis*), canine cyclic thrombocytopenia (*Anaplasma platys*), and canine babesiosis (*Babesia canis*) [24,25,27,28,29,30,31]. Additionally, *Hepatozoon canis*, another tick-borne hemoparasite affecting dogs, acquired from the ingestion of infected ticks, has been reported in Costa Rica and some of the Caribbean islands (e.g., Cuba, Grenada, St. Kitts, and Trinidad) [30,32,33,34,35,36]. Seroprevalence studies have shown exposure to spotted fever group *Rickettsia* in dogs from Panama and Costa Rica [37,38,39]. It should be noted that all of the aforementioned pathogens are transmitted or associated with the bite or ingestion of *Rhipicephalus sanguineus* sensu lato (s.l.) ticks. This exotic species was introduced to the region during the European colonization of the Americas and is now prevalent throughout the CAC [40]. Natural infections with *Rickettsia amblyommatis* and *Rickettsia rickettsii* have been reported in *R. sanguineus* s.l. [17]. Although there are reports of rickettsial infections in native ticks (including species of *Amblyomma*, *Haemaphysalis*, *Ixodes*, and *Ornithodoros*) [21,41,42], to date, there is no confirmation of these ticks being vectors of TBPs or cause of clinical cases in dogs from CAC, despite the reports of *E. canis* in *Amblyomma ovale* in Yucatán and *Rickettsia* sp. in *Ixodes affinis* [21,43]. In the case of the causative agent of Q fever, *Coxiella burnetii* (formerly *Rickettsia burnetii*), little is known about its epidemiology and distribution within the CAC. To date, a recent report of *C. burnetii* infecting *Amblyomma mixtum* ticks in Cuba [44], coupled with two older references, reported its detection in cattle and *Dermacentor nitens* ticks from Puerto Rico and Panama, respectively [45,46].

Regarding livestock, two exotic ticks (i.e., *Rhipicephalus microplus* and *A. variegatum*) are considered to be major threats to the industry [47,48]. *Rhipicephalus microplus*, a one-host tick, is the most economically important tick in the global livestock industry and is associated with the transmission of *Anaplasma marginale*, *Anaplasma centrale*, *Babesia bigemina*, and *Babesia bovis* [49]. In CAC, *R. microplus* is widely distributed and has been reported in different climatic conditions at altitudes varying from 0 to 2000 m [13,14,48,50]. *Amblyomma variegatum* is a three-host tick that, in addition to *E. ruminantium*, *R. africae*, and *R. conorii*, also transmits pathogens of the genera *Ehrlichia* and *Theileria* [10,51,52,53]. The bont tick was first introduced to Guadeloupe and was restricted to Guadeloupe, Antigua, and Martinique until the 1960s. However, it was widely disseminated among 18 Caribbean islands by the end of the 1980s, possibly spread by migrating cattle egrets (*Bubulcus ibis*) in the region [54]. Until now, the distribution of *A. variegatum* in the CAC is restricted to the Lesser Antilles [55]. However, expansions to other Caribbean islands and the continental landmass are expected due to favorable climatic conditions, increased introductions of immature ticks by cattle egrets, and increased movement of infested hosts [56]. 

Babesiosis is an important TBD caused by apicomplexan parasites of the genus *Babesia* and it affects several domestic and wild animals worldwide [57]. Canine babesiosis is one of the most frequently reported infections caused by *Babesia canis vogeli* and *Babesia gibsoni* in the CAC region. The prevalence of *Babesia* spp. has been reported to be as much as 20% using microscopic, molecular, and serological techniques in dog populations from Costa Rica, Grenada, Haiti, Nicaragua, St. Kitts and Nevis, and Trinidad and Tobago [32,34,58,59,60,61,62]. 

The potential tick vectors for these *Babesia* spp. include *R. sanguineus* s.l. and *Rhipicephalus turanicus* [63,64], with a potential wound to wound or dog-bite transmission of *B. gibsoni* [61,65]. Infections with *B. gibsoni* are usually acute and characterized by anorexia, fever, hepatomegaly, splenomegaly, and pallor, with carrier states common in recovered animals [65]. Other *Babesia* infections reported in mammalian hosts in the CAC include *B. gibsoni* and *Babesia vogeli* in cats [4,29,60], *Babesia canis rossi*, *Babesia vulpis*, *B. ovis*, *B. gibsoni*, *B. motasi*, and *Babesia caballi* in sheep and goats [64,66], *B. bovis*, *B. bigemina*, *B. vogeli*, and *B. gibsoni* in cattle [67,68,69,70,71,72,73,74], *B. bovis* and *B. bigemina* in buffalo [75], and *B. caballi* in horses [76,77,78]. Recently, *Babesia odocoilei* was reported for the first time from free-living *Ixodes* cf. *boliviensis* ticks in Panama [79].

Canine hepatozoonosis, caused by the apicomplexan *H. canis*, has also been reported frequently in the CAC and is transmitted by *R. sanguineus* s.l. [80]. Using microscopic, serological, and molecular techniques, up to 47.5% occurrence of *H. canis* has been reported in dogs from Aruba, Costa Rica, Cuba, Grenada, Haiti, Nicaragua, St. Kitts, and Trinidad [28,32,34,35,36,58,59,81]. Clinical manifestations include chronic weight loss, relapsing fever, usually unresponsive to babesicidal drugs and antibiotics and disturbed hematological parameters, including anemia and eosinophilia [82]. Moreover, *H. canis* has also been detected in spiny-tailed iguanas [83] and *Ixodes* cf. *boliviensis* ticks from Honduras and Panama, respectively [79].

Other tick-borne infections reported in the CAC include equine piroplasmosis caused by *Theileria equi* and *B. caballi*, transmitted by several tick species [12,64,67,77,78,84], as well as bovine theileriosis caused by *Theileria mutans* and *Theileria velifera* which are transmitted by *A. variegatum* [52]. Few other *Theileria* species with unknown pathogenicity have also been reported in cattle (*Theileria* sp. B15a) and sheep and goats (*Theileria* sp. NG-2013a, *Theileria* sp. *OT3, Theileria sp.* YW-2014) in the CAC region [66]. Despite the very high seroprevalence of equine piroplasmosis reported from a few regions of CAC with some evidence of the existence of endemic stability [77,78], there is a paucity of information on its molecular epidemiology and ecology.

Earlier studies reported relapsing fever (RF) spirochetes in cattle, horses, tamarins (*Saguinus geoffroyi*), opossums (*Didelphis marsupialis*), and armadillos (*Dasypus novemcinctus*) in Panama [85]. Further experimental infection trials with armadillos using a human isolate of these RF spirochetes resulted in parasitemic animals, thus indicating the significance of these mammalian hosts in the disease ecology [85]. The results of this, and other studies, highlight the importance of wildlife and tick vectors in the ecology and epidemiology of TBDs affecting human and animal health [86,87,88]. 

### 2.2. Ticks as Vectors of Human Pathogens of Bacterial and Protozoal Origin in the CAC

**Figure 1:**
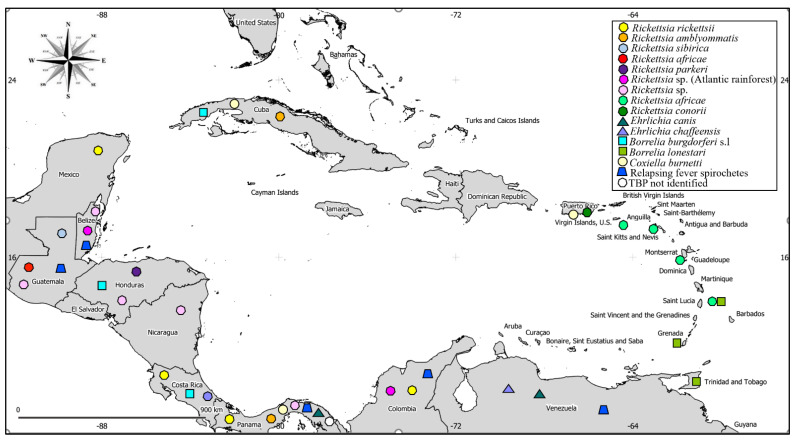
Geographic distribution of tick-borne pathogens (TBPs) affecting human health across countries in Central America and the Caribbean (CAC) region. The position of the dot within each country does not indicate the exact location of a pathogen. The map was generated using QGIS v3.18 (QGIS Development Team, 2021). Shapefile maps of the CAC region were kindly provided by geoMinds company (http://www.geominds.de/companyprofile.html, accessed 1 August 2021).

Among TBDs affecting humans living in or travelling to the CAC (Table 2), spotted fever group rickettsioses (SFGR) are most frequently reported [18,89,90,91,92]. The geographical distribution of major TBPs in the CAC is displayed in Figure 1. The pathogenic potential of other *Rickettsia* detected in ticks of the region is currently unknown (Table 3). Tick-borne relapsing fever (TBRF), southern tick-associated rash illness (STARI), and possible infections with Lyme borreliosis and canine ehrlichiosis have also been documented in humans [93,94,95,96,97]. With the exception of *Ornithodoros* spp. reported as known vectors of relapsing fever, other tick vectors are yet to be identified [93]. Relapsing fever has been previously reported in Colombia and Panama, and a new case was registered in a tourist in 2006 who visited Guatemala and Belize [98,99].

*Rickettsia rickettsii* is the most pathogenic and lethal member of the SFGR and the causative agent of Rocky Mountain spotted fever (RMSF) of humans. Within the CAC region, clinical cases of RMSF have been documented from Panama and Costa Rica, and the Caribbean coasts of Colombia and Mexico (Table 2) where *A. mixtum* and *R. sanguineus* s.l. are the main putative tick vectors [18,91,128,129]. *Rickettsia rickettsii* has been identified from nymphs of *A. mixtum*, *A. varium*, and *Haemaphysalis leporispalustris* from Costa Rica, and *D. nitens*, *A. mixtum*, and *R. sanguineus* s.l. from Panama [89,100,105,130,131]. Exposure to SFGR has also been reported in humans from Belize, Guatemala, Honduras, and Nicaragua as well as several Caribbean countries [17,51,101,124,132]. A less severe rickettsial disease, African tick-bite fever, caused by *R. africae*, has been reported in the eastern Caribbean [133]. This TBP was probably introduced into the Caribbean along with *A. variegatum*-infested cattle imported from Senegal more than 200 years ago. Although a small number of clinical human cases are reported, *R. africae* and its human-infesting vector, *A. variegatum*, are widespread in the Caribbean [103].

Since most TBDs have an incubation period ranging from a few days to weeks, information on the tick vector related to clinical cases is often unavailable. However, the possible vectors for specific TBDs can be identified by combining the tick bite history and clinical and laboratory data with epidemiological surveys of pathogen infections in questing ticks. For example, a traveler from Honduras developed a TBD and the laboratory results together with therapy response confirmed the diagnosis of rickettsial disease. Based on the tick research data available from the region, *Amblyomma maculatum* could be considered the vector and *Rickettsia parkeri* as the causative agent of the disease, despite the fact that this pathogen has not yet been identified in Honduras [110]. Additionally, reports of ehrlichiosis in Venezuela [134], Costa Rica [135], and Panama [112] could be attributed to zoonosis transmission from dogs to humans via infected *R. sanguineus* s.l. Furthermore, babesiosis should also be considered as a zoonoses risk in the CAC due to the serological findings of *Babesia* spp. in veterinary personnel, farmers, and soldiers from rural areas of Venezuela, and a severe case of babesiosis in an Austrian tourist who visited Nicaragua [136,137].

### 2.3. Tick-borne Viruses of Humans and Animals in the CAC

Currently, no published data exist on TBVs in Central America. However, Raza virus (from the Hughes virus group) has been isolated from *Ornithodoros* spp. collected from Raza Island, neighboring Mexico [138]. Several tick-borne viruses have been identified in ticks in the Caribbean region, including Hughes virus in *Ornithodoros capensis* complex (*Onithodoros denmarki* and *Onithodoros capensis*) from Trinidad [139] and *O. denmarki* in Cuba [140], Soldado virus in *O. capensis* complex in Trinidad [141], Estero real virus in *Onithodoros tadaridae* ticks in Cuba [142], and Wad Medani Virus in *Amblyomma cajennense* s.l. ticks from Jamaica [143]. African swine fever virus, which was endemic to Hispaniola (Haiti and the Dominican Republic) and Cuba in the 1980s, was also detected in *Ornithodoros* ticks [144,145]. It should be noted that even though the African swine fever virus was not identified in any of 350 *Onithodoros puertoricensis* ticks collected in the Dominican Republic and Haiti, ticks were able to acquire and transmit this virus transstadially and transovarially under laboratory conditions [146,147]. 

Sameroff et al. [148] described the virome of 638 ticks, including *R. microplus* (*n* = 320), *R. sanguineus* s.l. (*n* = 300), and *A. ovale* (*n* = 18) collected in Trinidad and Tobago in 2017 and 2018. Sequences representing nine viruses were identified, including five novel species within Tymovirales, Bunyavirales, Chuviridae, Rhabdoviridae, and Flaviviridae, namely Trinbago virus (Flaviviridae) in three ticks species (*R. microplus*, *R. sanguineus* s.l., and *A. ovale*), two brown dog tick phleboviruses (Phenuiviridae), one Wuhan mivirus (Chuviridae) in *R. sanguineus* s.l. and *R. microplus*, one brown dog tick mivirus (Chuviridae) in *R. sanguineus* s.l., one Blanchseco virus (Rhabdoviridae) in *A. ovale*, two Jingmen tick viruses (C and AS), one Lihan tick virus in Trinidad, and one cattle tick tymovirus-like virus (unclassified) in *R. microplus* [148]. 

Similarly, using the next-generation sequencing approach, Gondard et al. [149] analyzed the virome of 578 *A. variegatum* and *R. microplus* ticks collected in Guadeloupe and Martinique islands in 2014–2015. They reported sequences of viruses infecting plants or restricted only to arthropods and included four viruses either belonging to arboviruses (Flaviviridae and Peribunyaviridae-related viruses) or viruses of unknown pathogenic potential to vertebrates (Chuviridae-related viruses). In this study, Karukera virus was detected in 23% of *A. variegatum* ticks from Guadeloupe, Wuhan Tick Virus 2 and Lihan Tick Virus in 63–94% of *R. microplus* and 11–12% *A. variegatum* in both Islands, and Jingmen Tick Virus mainly in 24–74% of *R. microplus* from both islands [149].

A diverse wildlife population exists in the CAC region which may host a number of tick species with the potential to spread pathogens to domestic animals and humans. However, the role of these ticks in transmitting viruses in wildlife remains understudied. A recent study in Trinidad reported three novel viruses, classified as Granville quaranjavirus (GQV) (Orthomyxoviridae), *Amblyomma dissimile* mivirus (ADM) (Chuviridae), and a tick-borne tetravirus-like virus (Tetraviridae) [150]. Of note, GQV genome bears sequence homology to known zoonotic influenza virus and thogotovirus [151]. Quaranjaviruses have also been associated with unexplained febrile illness in children and cyclic avian mortalities [152,153]. Due to the scarcity of information on TBVs in the CAC, further research is warranted to elucidate the pathogenic potential of these TBVs for the human and animal populations. 

### 2.4. Tick-Borne Co-Infections of Humans and Animals in the CAC

Co-infections due to TBPs are well-characterized in the Caribbean region, notably in dogs. For example, a recent study in Grenada analyzed 455 blood samples from dogs (*n* = 358), sheep and goats (*n* = 65), and cattle (*n* = 32) using a new multiplex real-time PCR [154]. Although co-infections were not detected in ruminants, 1.96% (7/358) dog samples were co-infected with two pathogens of the family Anaplasmataceae (i.e., *E. canis* and *Ehrlichia chaffeensis* in two dogs, *E. canis* and *A. platys* in four dogs, and *A. platys* and *Ehrlichia ewingii* in one dog). A previous study on the same island found that dogs were exposed to both *E. canis* and *A. platys* (6.0%) [155]. Earlier, a serological study on 73 dogs from Grenada detected co-infections with *E. canis* and *A. platys* in four dogs, *A. platys*/*H. canis, E. canis/B. canis vogeli*, or *E. canis/H. canis* in one dog, and a triple infection of *E. canis, A. platys*, and *B. canis vogeli* in one dog [63].

In Haiti, co-infection in dogs with two or more TBPs was detected using serology (20.0%) and molecular methods (10.6%) [28]. The most prevalent co-infection involved *Dirofilaria immitis* and *B. vogeli* (3.4%) followed by *D. immitis* and *E. canis* (1.9%), *D. immitis* and *H. canis* (1.4%), *E. canis* and *H. canis* (1.4%), *H. canis* and *Acanthocheilonema reconditum* (1.0%), *D. immitis* and *A. platys* (0.5%), *E. canis* and *B. vogeli* (0.5%), and *H. canis* and *A. platys* (0.5%) [28]. In Puerto Rico, 25.2% (31/123) of dogs were found co-infected with two or more pathogens (including *D. immitis*, *E. canis*, and *Anaplasma phagocytophilum*) through ELISA [156]. In St. Kitts, co-infections were detected in 15.0% of dogs with the most prevalent association between *E. canis* and *Babesia* spp. (10.9%) followed by *E. canis* and *H. canis* (3.6%) and *B. canis vogeli* and *B. gibsoni* (0.9%) [30].

In Cuba, co-infections with three hemoparasites (*B. bigemina*, *A. marginale*, and *B. bovis*) were found in 12.0% of water buffaloes, with co-infections of *B. bovis* and *A. marginale* being the most common (26.0%) followed by *B. bovis*/*B. bigemina* (20.0%) and *A. marginale*/*B. bigemina* (24.0%), suggesting the potential positive interaction between these pathogens [157]. Similarly, another study from Cuba reported co-infections with *B. caballi* and *T. equi* in 20.0% of horses tested [76]. Rodriguez et al. [158] also reported *A. ovis* and *B. ovis*, *A. ovis* and *E. ovis*, and *A. ovis* and *B. motasi* co-infections in sheep through microscopic examination of blood smears.

In Guadeloupe and Martinique, co-infections were not found in *A. variegatum* ticks, while *R. microplus* ticks presented co-infections with two to three viruses [149]. In Guadeloupe, 49.0 and 37.0% of *R. microplus* ticks were infected with the Wuhan Tick Virus 2/Lihan Tick Virus and Wuhan Tick Virus 2/Jingmen Tick Virus/Lihan Tick Virus, respectively. In Martinique, Wuhan Tick Virus 2/Jingmen Tick Virus/Lihan Tick Virus triple infection represented up to 76% of positive *R. microplus* ticks, while double infections with Wuhan Tick Virus 2/Lihan Tick Virus in 14% of ticks [149]. This study also investigated bacterial and parasitic co-infections and reported co-infections with two, three, and four/five pathogens in more than 40%, less than 10.0%, and around 1.0% of ticks, respectively [149]. Moreover, another study has demonstrated associations of several pathogens, mainly with *R. africae* in *A. variegatum* and *R. africae* or *T. velifera* in *R. microplus* [70]. 

Reports of co-infections in humans in the Caribbean are rare. However, a case of a young veterinarian co-infected with *A. platys*, *Bartonella henselae*, and *Candidatus Mycoplasma haematoparvum* has been reported in Grenada [159]. Infection in this case could have resulted from the profession-related risk of being exposed to a number of domestic animals and wild game, thus increasing the exposure to tick bites and thus the probability of multiple-pathogen infections.

Co-infections have been less commonly reported in animals in Central America. A seroprevalence study in Costa Rica has revealed that 10.3% of dogs were co-infected with *A. phagocytophilum* and *A. platys* [59,160]. Another study using PCR reported that 12% of dogs were co-infected with two pathogens, including *E. canis* and *B. vogeli* (5.5%), *E. canis* and *A. platys* (2.7%), *E. canis* and *H. canis* (2.0%), *A. platys* and *B. vogeli* (1.4%), and *A. platys* and *H. canis* (0.7%) [34]. A commercial serologic test revealed that three of twenty-two dogs (13.6%) in Panama showing hematological signs characteristic of TBDs were co-infected with *A. platys*/*A. phagocytophilum* and *E. canis/E. chaffeensis* [25]. Such findings highlight the need for reliable diagnosis of TBPs as a pre-requisite to effective treatment of infected hosts (animals and humans) [25]. Bacterial co-infections reported in ticks included *Rickettsia bellii* and Candidatus *Rickettsia colombianensi* in *A. dissimile* collected from *Iguana iguana* in Panama and *Rickettsia* sp. and *A. phagocytophilum* in free-living *Ixodes tapirus* females [79,127].

## 3. Diagnostic Tools for TBPs in the CAC

In the CAC region, six major animal TBPs, including *A. marginale*, *E. ruminantium*, *B. bovis*, *B. bigemina*, *B. caballi*, and *T. equi*, are actively monitored due to their socioeconomic impact [62,77,157]. However, despite the high burden of TBDs reported in the region, only a few studies have assessed the diversity of tick species and the incidence of TBDs in humans [106,116,132,135]. In the last few decades, some epidemiological studies have been conducted in companion animals, mainly dogs and cats from the CAC region, using serological methods such as the commercial rapid ELISA SNAP^®^ 4Dx^®^ Plus, and immunochromatography tests SNAP 3DX^®^ and SNAP 4DX^®^ [28,59,160,161]. These serological assays were produced by IDEXX Laboratories (Westbrook, Maine, USA) and are designed to detect antibodies against *A. phagocytophilum*/*A. platys*, *Borrelia burgdorferi* s.l., and *E. canis*/*E. ewingii*, as well as antigens of *D. immitis* [162]. Globally, serological methods are employed extensively in epidemiological studies to detect subclinical infections. However, false positive and negative results are commonly observed in such serological tests due to cross-reactivity, poor specific immune response, and an inability to detect antibodies in carriers [163]. In addition, serological tests are not useful for detecting current or early acute infections (because of delayed seroconversion), parasite clearance post-treatment, and diagnosis of pathogens in tick samples [164]. 

To overcome the aforementioned drawbacks, a variety of molecular methods have been employed in the CAC region for the surveillance of TBPs. These include conventional PCR (cPCR), nested PCR (nPCR), reverse line blot (RLB), quantitative fluorescence resonance energy transfer (FRET) PCR, multiplex TaqMan^®^ real-time PCR, and multiplex PCR coupled with oligonucleotide probe based multi-analyte profiling bead (xMAP) [29,62,154,165]. Although molecular assays employed for the detection of TBPs have yielded higher sensitivities and specificities than serological detection methods, the main disadvantage of these approaches is that a limited number of pathogens can be tested simultaneously. Therefore, advanced and extended surveillance tools are needed to assist in large-scale epidemiological studies. In this regard, Gondard et al. [70] implemented a high-throughput microfluidic real-time PCR for the screening of several important and neglected TBPs, including bacteria (five genera and twenty-five species) and parasites (three genera and seven species), potentially circulating in ticks in the CAC region. This assay utilized the BioMark^TM^ real-time PCR system which can perform parallel real-time PCRs using either 96.96 chips or 48.48 chips, resulting in either 9,216 or 2,304 individual reactions, respectively [166]. Recently, the microfluidic real-time PCR system was successfully employed for screening of TBPs in human blood samples and *Ixodes ricinus* ticks from Serbia [167]. Thus, this surveillance method represents a major advancement for the large-scale epidemiological studies in the CAC region, with a focus on developing efficient tick control programs and prevention strategies against (re)emergence of TBDs with a ‘One Health’ approach.

## 4. Current Situation and Perspective of Surveillance Programs in the CAC

In 1994, the Caribbean *Amblyomma* Program (CAP), led by the Food and Agriculture Organization (FAO) of the United Nations, was launched to facilitate the active surveillance and eradication of *A. variegatum*. A total of 12 islands of the Eastern Caribbean, US territories in the Caribbean, and the French West Indies participated in this program [168]. Stakeholder awareness and training activities were vital for the success of CAP and resulted in empowering livestock owners with the responsibility of treating their animals [169]. Additionally, a regional database “TickINFO” was developed to collect and analyze the results of the surveillance activities. Despite the real success of CAP, and the attainment of tick-free certification for seven countries, several issues were highlighted by the coordination team in 2007 [170]. Some technical, scientific (e.g., technical design, methodology), and ecological factors (e.g., drought and tick biology) were responsible for heterogeneous results in different territories [170]. Furthermore, administrative (i.e., multi-organizational framework), management, and financial issues (e.g., multi-sources with separate management and non-continuous funds) impacted negatively on the implementation of CAP.

Between 2013 and 2015, CIRAD, Guadeloupe, in collaboration with the Veterinary Services of St. Vincent and the Grenadines and the US Virgin Islands, spearheaded a regional project, ResisT [171], which was focused on acaricide resistance and improved surveillance and control of ticks (*A. variegatum* and *R. microplus*) and TBDs (heartwater, anaplasmosis, and babesiosis) in ruminants in the Caribbean. Partners included the Veterinary Services of Antigua and Barbuda, Dominica, Guadeloupe, Martinique, St. Kitts and Nevis, Saint Lucia, and Martinique. However, financial challenges and the lack of human resources from some of the stakeholder territories limited the full execution of ResisT.

Tick samples from Guadeloupe and Martinique were analyzed revealing the presence of seven TBPs, *R. africae*, *E. ruminantium*, *A. marginale*, *B. bigemina*, *B. bovis*, *T. mutans*, and *T. velifera* [70]. Nevertheless, this survey did not imply the existence of an active surveillance system in these countries but highlighted the novel diagnostic tools implemented. Indeed, surveillance is defined as a systematic and continuous or repeated collection, analysis, and dissemination of animal health or welfare related data [172] and should be clearly differentiated from sporadic surveys [173].

The experience of the CAP provided a wealth of knowledge on the implementation of surveillance systems at a regional scale with the opportunities and pitfalls. The importance of regional coordination and the involvement of each territory are mandatory in the success of any surveillance program. The history of regional collaboration and lessons learnt from the CAP have contributed to the formalization of the regional network for animal health and veterinary public health, CaribVET [174]. Since its first Steering Committee meeting in 2006, CaribVET has focused on coordinating activities dedicated to improving animal health through a regional strategy developed by the chief veterinary officers (CVOs) of the 34 Caribbean territories with the support of 14 international agencies, and academic and research organizations. Epidemiological surveillance of vectors and vector-borne diseases (VVBD) such as ticks and TBPs is a transversal task tackled by the Epidemiology and the VVBD working groups within the network. Aside from this regional approach, the network aims to increase the knowledge with its research activities and develop innovative tools to enhance the technical capacities of its members and to optimize the surveillance and control of diseases in the region.

## 5. A ‘One Health’ Dimension to TBDs in the CAC

Knowledge of the ‘One Health’ tenets is essential for veterinarians and physicians. Over the past three decades, emerging zoonotic infectious diseases have implicated wildlife as the main pathogen reservoirs driven by changes in the ecosystem due to human activity [1,175,176,177]. In the context of ticks and TBDs, the ‘One Health’ approach will aid in improved diagnosis, the acceleration of treatment decisions, and the implementation of prevention and control protocols. Introduction to ‘One Health’ concepts should therefore be implemented during the early years of training of medical and veterinary students since both groups are rarely provided with opportunities for inter-professional learning during their coursework and clinical training [178,179]. Furthermore, overcoming the hurdles of interdisciplinary communication and collaboration among physicians and veterinarians is important in order to confront the health problems associated with ticks and TBDs affecting humans and animals.

One possible solution is continuous education and training on TBDs to medical and veterinary professionals in a common setting. An estimation of the current situation is necessary to assess the knowledge of health service providers in this area. In the CAC region, only one knowledge survey about TBD (Lyme disease) and physicians has been reported from Cuba [180]. Although Lyme disease has not been officially reported on this island, clinical and serological evidence suggests its presence in the human population [116,181]. A survey of medical doctors on their knowledge of the disease revealed that 70% had some knowledge of Lyme disease while 78.6% of them identified it as a TBD, 82% identified it as a bacterial etiology, 46% recognized at least one of its clinical manifestations, and only 36% knew about the required laboratory testing [180]. As such, continuing education is needed to equip medical personnel with knowledge to diagnose and treat Lyme disease and other TBDs [180]. This situation is not exclusive for Cuba as similar results have been reported in other regions of the world where TBDs are more prevalent [182]. Surveys of knowledge, attitudes, and practices about specific or general TBDs have been conducted with physicians, veterinarians, and students of both professions [182,183,184]. The main knowledge gaps identified were non-recognition of diseases transmitted by tick bites, their clinical manifestations and severities, frequency of occurrence in daily practice, the incorrect identification of the names of TBDs and TBPs, the lack of information about tick infestation (areas at risk), correct removal and preventive and treatment measures, the inappropriate serology test requests for tick bites, the use of tick analysis for diagnostic purposes, and the use of different drugs for treatment of children and adults [182,183,184].

Similar qualitative studies are necessary for the CAC for gleaning and disseminating information among medical personnel in the territories due to the rich diversity of tick species and TBPs in this region [4,59,109]. As such, there is a need to educate/train physicians, veterinarians, and medical and veterinary students about the existence of TBDs and the management of human and animal cases in the CAC. This awareness can be improved by increased problem-based learning modules that develop the capacity to apply acquired knowledge to solve problems and more cross-disciplinary teaching activities, and implementation or continuation of information campaigns, all from a ‘One Health’ perspective. Moreover, popular knowledge can be enriched with scientific data which would improve the perception of these diseases among the general public [185,186].

## 6. Treatment Protocols Used for Animals and Humans with TBDs

Antimicrobial chemotherapy for rickettsial diseases in humans is achieved mainly by the oral administration of tetracycline and doxycycline [187]. The latter is also used for the treatment of companion animals infected with RMSF rickettsiae and is available in oral and intravenous formulations [188]. Treatment guidelines in some countries of the CAC, for example, Panama, and those published by the Centers for Disease Control and Prevention (CDC) of the USA, recommend the use of doxycycline in human patients of all ages with suspected rickettsial infections [187]. Álvarez-Hernández et al. [189] identified two main factors that influenced the clinical outcomes in patients with rickettsial infection in America. These included early diagnosis and timely initiation of antibiotic treatment (within 3–5 days from the first onset of symptoms) [189]. The initiation of specific treatment should not be subject to laboratory confirmation and must be fully supported by the empirical suspicion of the disease. Another element that could reduce the mortality associated with severe rickettsial illness in animals and humans is the use of intravenous preparations of doxycycline. This is especially important in cases where vomiting reduces the bioavailability of the drug. Blanton et al. [190] demonstrated that parenteral application of tigecycline was effective for the treatment of *R. rickettsii* infection in animals with RMSF and could possibly be used in cases where intravenous doxycycline formulation is not available.

Doxycycline is recommended as the first drug of choice for the treatment of most TBDs and chloramphenicol as the alternative in cases of doxycycline allergy [164]. A single 200 mg oral dose of doxycycline was superior over a 5–10 day course of chloramphenicol in reducing time under fever for mild cases of other rickettsial infections, such as murine typhus caused by *Rickettsia typhi* and transmitted by fleas [191]. Chloramphenicol may be an alternative treatment for RMSF but not for human granulocytic anaplasmosis (HGA) or human ehrlichiosis [164]. Considering that chloramphenicol is associated with adverse hematologic effects, tetracycline has been used as an alternative for the treatment of RMSF with good results [164]. In cases of patients with severe doxycycline allergy, or who are pregnant, the USA guideline recommends rifampin as an alternative to doxycycline for the treatment of HGA or human ehrlichiosis [164]. However, no report was found of rifampin usage for the treatment of HGA or human ehrlichiosis in the CAC. 

The risk of rickettsioses in the general population is low compared to more common diseases and therefore most physicians are not aware of the procedures to follow to prevent and/or treat these diseases [192]. For example, in a rural village in western Panama, it was reported that seven individuals (3–20 years of age) with no history of tick bites were affected by severe RMSF and presented with non-specific symptoms such as generalized exanthema, diarrhoea, vomiting, and headaches [91]. Late diagnosis and treatment resulted in the recovery of two patients after treatment with doxycycline, one recovery without doxycycline, and four died [91]. Another study reported the death of a teenager in Ciudad de Panamá, Panama, after an acute and lethal case of rickettsiosis [192]. In western Nicaragua, Reller et al. [110] reported several cases of acute rickettsial infections that were unsuspected and untreated, despite the availability of doxycycline. The authors mentioned challenges in distinguishing rickettsial infections from other febrile diseases. In 2007, there was a rickettsial febrile illness outbreak in Guatemala and patients were treated with a variety of antimicrobials, including amoxicillin, penicillin, erythromycin, trimethoprim, and doxycycline [101]. Among the 17 cases registered in this outbreak, two patients died despite being treated with one dose of doxycycline and amoxicillin, respectively, whereas two other patients recovered without any medication [101]. It is noteworthy that in none of these two outbreaks in Panama and Guatemala, did the patients report being bitten by ticks, while six patients from the Nicaragua study reported flea bites [101,110]. These examples show the importance of early diagnosis and treatment as well as the role of physician awareness for the reduction of mortality due to TBDs in endemic and non-endemic areas.

Concerning Lyme borreliosis, oral amoxicillin and doxycycline are considered equally efficient for the first stage of the disease (i.e., erythema migrans), while intravenous administration of ceftriaxone is recommended in cases of neuroborreliosis [193,194] and Lyme carditis [195]. However, it is important to mention that Lyme borreliosis is not commonly reported in the CAC [97,100]. Full recovery was also observed in a patient treated with ceftriaxone (a third-generation cephalosporin) with a case of neuroborreliosis in Honduras [97]. Although the data about cases of Lyme borreliosis incidence and therapeutic outcomes in this region are scarce, from these reports we can assume that pathogenic *Borrelia* spp. in the CAC show similar antibiotic susceptibility to antimicrobial therapy as seen in Europe and North America.

## 7. Concluding Remarks

Several ticks and TBPs are present in the CAC, posing a risk to human and animal health. Some of these pathogens are restricted to transmission cycles involving mostly domestic animals (e.g., *E. canis*) and wildlife and/or livestock (e.g., *E. ruminantium*), while others are comparatively highly prevalent in humans (e.g., SFGR). *A. mixtum*, *A. variegatum*, and *R. sanguineus* s.l. are the important vectors in the region due to their potential for transmitting zoonotic pathogens. Although information about TBPs affecting domestic animals is available, little is known about the situation for diseases affecting humans. More research is needed to assess the impact that co-infections reported in the region have on the serological and molecular diagnosis of TBDs. 

The chains of infection of TBPs frequently involve multiple contributors, including different life stages of the vector (questing or feeding larvae, nymphs, and adults ticks), wildlife, domestic animals, livestock, and humans. However, most of the epidemiological studies in the region have explored only one of these elements in a localized environment. For example, if the prevalence of *Borrelia* in dogs is studied, other animal species, humans, and ticks (questing and feeding ticks) in the same environment are not tested simultaneously. This fact is important with the recent finding of *B. burgdorferi* group in free-living *I*. cf. *boliviensis* in Panama, which is a synanthropic species that has been reported parasitizing dogs and humans in the highlands of that country and Costa Rica [79].

The role of wildlife in the transmission of TBPs is poorly understood in the region. For instance, in recent years, several species of *Rickettsia*, *Anaplasma*, *Babesia*, and *Hepatozoon* have been reported, both in ticks and ectothermic and endothermic vertebrates in several CAC countries (Table 3). Further studies are needed to identify and elucidate the connection between wildlife reservoirs and the emergence of TBPs along with spillover events from wildlife to domestic animals, livestock, and humans. Additionally, the impact that TBPs circulating in domestic animals and livestock can have on wildlife should also be studied. Thus, the threats that TBPs affecting livestock posed on biodiversity should also be a focus of research. 

In conclusion, there are major gaps in our understanding of TBPs prevalent in CAC and only the use of the ‘One Health’ approach involving the collaboration of veterinarians, physicians, and researchers can overcome this limitation. Such studies can include the sampling of animals, ticks, and humans in a defined location, combined with high-throughput molecular detection of pathogens as well as serology [7]. A positive thrust towards the implementation of the ‘One Health’ approach in the CAC with respect to ticks and TBPs/TBDs is the availability of state-of-the-art diagnostic tools that have been developed and used for their detection and identification in the region. Although there is always a room for the improvement, regional authorities are developing human resources specialized in ticks and TBPs and the know-how to use advanced techniques to study pathogen epidemiology.

## Figures and Tables

**Table 1:** Ticks and tick-borne pathogens of animals in the Central America and the Caribbean region.

Pathogen	Disease	Tick Vector(s)	Vertebrate Hosts	Countries ^a^	Reference(s)
*B. burgdorferi* group ^b^	Lyme disease	Unknown	Cat Dog	Hon CR	[19] [20]
*A. marginale*	Anaplasmosis	*R. microplus*	Cattle	An, Ba, Cu, D, DR, Gr, Gp, H, J, Ne, SK, Ma, M, PR, SM, SV, Tr	[4]
*A. odocoilei*	Unknown	Unknown	Deer	Mex	[21]
*A. phagocytophilum*	Granulocytic anaplasmosis	Unknown	Cattle Deer	PR Mex	[4] [22]
*A. platys*	Canine cyclic thrombocytopenia	*R. sanguineus* s.l.	Dog	Gr, Pan	[23]
*E. canis*	Tropical canine pancytopenia	*R. sanguineus* s.l.	Dog	Ar, BWI, CR, D, ES, Gr, H, PR, SK, NE, M, Nic, Pan, Tr, TC	[4,23,24,25]
*E. canis*	Tropical canine pancytopenia	*A. ovale*	Dog	Mex	[21]
*E. chaffeensis*	Unknown	*Amblyomma* spp.	Deer	Mex	[22]
*E. ruminatium*	Heart water disease	*A. variegatum*	Cattle	An, D, Ma, M, N, SK, SL, VI	[4]
*B. bigemina*	Babesiosis	*R. microplus*	Cattle	An, Ba, Cu, D, DR, G, Gp, H, J, Ma, Mo, Ne, PR, SK, SL, SM, SV, Tr	[4]
*B. bovis*	Piroplasmosis	*D. nitens*	Cattle	An, Ba, Cu, D, DR, G, Gp, H, J, Ma, M, Ne, PR, SK, SV, Tr	[4]
*B. caballi*	Piroplasmosis	*D. nitens*	Equids	G, Gp, Ma, M, KN, Tr	[4,26]
*B. canis*	Canine babesiosis	*R. sanguineus* s.l.	Dog	Mo	[4]
*B. gibsoni*	Babesiosis	*R. sanguineus* s.l. *R. turanicus* ^c^	Several mammals	D, Ne, SK	
*B. rossi*	Babesiosis	*R. sanguineus* s.l. *R. turanicus* ^c^	Goat	Mo	
*B. vogeli*	Babesiosis	*R. sanguineus* s.l. *R. turanicus* ^c^	Goat	D, Gp, Ma, Ne, Mo, SK, Tr	
*B. vulpes*	Babesiosis	Unknown	Goat	Mo	
*H. canis*	Hepatozoonasis	*R. sanguineus* s.l.	Dog	Ar, CR, Gp, H, KN, T	
*T. equi*	Theileriosis	*D. nitens*	Livestock	D, Ne, SK, Tr	
*T. mutans*	Theileriosis	*A. variegatum*	Cattle	Cu, Gp, Ma	
*T. parva*	Theileriosis	*A. variegatum*	Cattle	Gp	
African swine fever virus	African swine fever	*Ornithodoros* spp.	Cattle	Cu, DR, H	
Hugues Virus	Not named	*O. capensis* group	Seabirds	Cu, Tr	

^a^ An: Antigua, Ar: Aruba, Ba: Barbados, Be: Belize, BWI: British West Indies, Col: Colombia (Caribbean coast), CR: Costa Rica, Cu: Cuba, DR: Dominican Republic, D: Dominica, ES: El Salvador, Gr: Grenada, Gp: Guadeloupe, Gua: Guatemala, Haiti: H. Hon: Honduras, J: Jamaica, Ma: Martinique, Mex: Mexico (Yucatán), Mo: Montserrat, Ne: Nevis, Nic: Nicaragua, Pan: Panama. PR: Puerto Rico, SK: St. Kitts, SL: St. Lucia, STM: St. Martin, Tr: Trinidad and Tobago, TC: Turks and Caicos, USVI: U.S. Virgin Islands. ^b^ Serological suggestion of potential autochthonous cases of Lyme disease, must be confirmed in CAC. ^c^ Presence of *R. turanicus* en CAC must be confirmed.

**Table 2:** Ticks and tick-borne pathogens of humans in the Central America and the Caribbean region.

Pathogen	Disease(s)	Tick Vector(s)	Countries ^a^	Reference(s)
*R. rickettsii*	RMSF	*A. mixtum* (*A. cajennense*) *A.* cf. *parvum* *A. varium* *D. nitens*	Pan, CR, Col, Mex	[18,100,101]
		*H. leporispalustris*	CR	[102]
		*R. sanguineus* s.l.	Pan	[89,90]
*R. sibirica*	Currently no reported human clinical cases	*A. mixtum* (*A. cajennense*)	Gua	[101]
*R. africae*				
*R. africae*	African tick-bite fever	*A. variegatum*	An, D, Gp, Ma, Mo, Ne, SK, SL, USVI	[51,103]
*R. amblyommatis*	Subclinical infection	*A. mixtum* (*A. cajennense*)	Cu	[104]
	RMSF		Pan, CR, ES, Hon	[27,100,105,106,107]
		*R. sanguineus* s.l.	CR, Pan	[100]
		*A. ovale*	Pan, CR	[100,105,108]
		*D. nitens*	CR	[100]
*Rickettsia* sp. strain Atlantic rainforest	Spotted fever	*A. ovale*	Be	[109]
*R. parkeri*	Erythema, induration and necrosis at the site of the tick bite, headache, fever, diarrhea, flu-like symptoms	*A. maculatum*	Hon	[92,110]
	*R. parkeri* rickettsiosis	*A. maculatum* *A. ovale*	Be	[109,111]
*R. conorii*	Mediterranean spotted fever	*A. variegatum*	Gp	[12]
*Rickettsia* sp.	Rickettsiosis	Unknown	Be, Gua, Nic, Hon, Pan	[12]
*E. canis*	Headache, myalgia, generalized erythema, elevation of transaminases, thrombocytopenia, hyponatremia, hypotension	*R. sanguineus* s.l.	Pan, Ve	[112,113]
*E. chaffeensis*	Non-specific symptoms (fatigue, arthralgia, myalgia)/subclinical infection	Vector tick species not identified	CR, Ve	[114,115]
*B. burgdorferi* s.l.	Lyme borreliosis	Vector tick species not identified	CR, Cu, Hon	[75,94,97,116]
*B. lonestari*	STARI	*Amblyomma* spp.	SL, Tr, Gr	[96]
Relapsing fever spirochetes	TBRF	*O. talaje*	Pan, Gua	[99,117]
		*O. puertoricensis*	Pan	[99,118,119]
		*O. rudis*	Pan, Ve, Co	
		Vector tick species not identified	Gua, Be	[95]
TBP not identified	Paralysis	*A. ovale*	Pan	[120]
*C. burnetii*	Q fever	*A. mixtum* *D. nitens*	Cu, Pan, PR	[44,45,46]

^a^ An: Antigua, Be: Belize, Col: Colombia (Caribbean coast), Ve: Venezuela (Caribbean coast), CR: Costa Rica, Cu: Cuba, D: Dominica, ES: El Salvador, Gr: Grenada, Gp: Guadeloupe, Gua: Guatemala, Hon: Honduras, Ma: Martinique, Mex: Mexico (Yucatán), Mo: Montserrat, Ne: Nevis, Nic: Nicaragua, Pan: Panama. PR: Puerto Rico, SK: St. Kitts, SL: St. Lucia, USVI: U.S. Virgin Islands.

**Table 3:** Bacterial and protozoal organisms detected in ticks from the Central America and the Caribbean region.

Microorganism	Ticks Host(s)	Countries ^a^	Reference(s)
Anaplasmataceae	*D. nitens*	Cu	[4]
*A. phagocytophilum*	*I. tapirus*	Pan	[79]
*R. africae*	*A. variegatum*	An, Gp, Ma, Mo, Ne, SK, SL, USVI	[51,103,121,122,123]
*R. amblyommatis*	*A. auricularium* *A. mixtum (A. cajennense)* *A. geayi* *A. longirostre* *A*. *maculatum* *A. mixtum* *A. ovale* *A. pacae* *A.* nr. *parvum* *A. mixtum (A. cajennense)* *A. varium* *D. nitens* *H. juxtakochi* *R. sanguineus* s.l.	Pan Be CR, Pan CR, Hon, Pan Be CR, Cu, ES, Hon, Pan CR Be, Pan ES Nic Pan CR, Pan Pan CR, Pan	[27,124,125,126]
*R. asemboensis*	*A. ovale* *R. microplus*	CR	[124]
*R. bellii*	*A. dissimile* *A. ovale* *A. rotundatum* *A. sabanerae*	Be, Pan ES Pan ES, Pan	[124]
*R. felis*	*A. mixtum*	Pan	[124]
*R. rhipicephali*	*D. latus*	CR	[124]
***Candidatus* species**			
*Ca*. “*Rickettsia colombianensi*”	*A. dissimile* *A. sabanerae* *A. scutatum*	CR, Pan CR ES	[124,125]
*Ca*. “*Rickettsia nicoyana*”	*O. knoxjonesi*	CR	[124]
**Non-described *Rickettsia***			
*R.* st. Aragoi	*A. triste*	Nic	[124]
*R.* st. Atlantic Rainforest	*A. ovale*	Be	[124]
*R.* st. IbR/CRC	*I.* cf. *boliviensis*	CR, Pan	[124]
*Rickettsia* endosymbiont *Ixodes*	*I. affinis*	Mex, Be, Pan	[126]
*Rickettsia* endosymbiont *Ixodes*	*I. tapirus*	Pan	[79]
*Rickettsia* spp.	*A. ovale* *R. sanguineus* s.l.	Be	[124]
*Rickettsia* sp.	*I.* nr. *minor*	CR	[124]
*Rickettsia* C325	*A. longirostre*	Pan	[126]
*Rickettsia* closely related to *R. africae*	*A. ovale*	Nic	[124]
*Rickettsia* closely related to *R. raoultii*	*A. geayi*	Pan	[127]
*Rickettsia* closely related to *R. tamurae*	*A. dissimile*	Pan	[125]
*B. burgdorferi* group	*I.*cf. *boliviensis*	Pan	[79]
*B.* cf. *odocoilei*	*I.* cf. *boliviensis*	Pan	
*Hepatozoon* sp.	*I.* cf. *boliviensis*	Pan	

^a^ An: Antigua, Be: Belize, Col: Colombia (Caribbean coast), CR: Costa Rica, Cu: Cuba, ES: El Salvador, Gp: Guadeloupe, Gua: Guatemala, H: Haiti, Hon: Honduras, Ma: Martinique, Mex: Mexico (Yucatán), Mo: Montserrat, Ne: Nevis, Nic: Nicaragua, Pan: Panamá, PR: Puerto Rico, SK: St. Kitts: SK, SL: St. Lucia, SM: St. Martin, USVI: U.S. Virgin Islands.

## Data Availability

Not applicable.
